# Human T-cell leukemia virus type 1 (HTLV-1) Tax oncoprotein induces DNA damages through Activation-Induced cytidine Deaminase (AID)

**DOI:** 10.1186/1742-4690-11-S1-O45

**Published:** 2014-01-07

**Authors:** Aurélien Riquet, Sébastien Chevalier, Julien Villaudy, Louis Gazzolo, Jean-Pierre Vartanian, Renaud Mahieux, Madeleine Duc-Dodon, Nathalie Bonnefoy

**Affiliations:** 1 Université de Lyon, Lyon, France; 2 Centre International de Recherche en Infectiologie INSERM U1111 - CNRS UMR5308, Université de Lyon, Ecole Normale Supérieure de Lyon, France; 3 Laboratoire de Biologie Moléculaire de la Cellule, UMR5239 CNRS, Ecole Normale Supérieure de Lyon, Lyon, France; 4 Unité de Rétrovirologie Moléculaire, Institut Pasteur, Paris, France; 5 Institut de Recherche en Cancérologie de Montpellier, Inserm U896 - Université Montpellier 1 - CRLC Val d'Aurelle, Montpellier, France

## 

How T cells are transformed by HTLV-1 is still unclear, but it is well accepted that the viral oncoprotein Tax is associated with genomic instability of infected cells. Tax has recently been shown to directly induce, in T cells, the expression of AID (Ishikawa C *et al.*, Carcinogenesis, 2011), a cytidine deaminase whose physiologic expression is usually restricted to B cells, in which it initiates class-switch recombination and somatic hypermutations to reshape the primary antibody repertoire after antigen encounter. It is also well established that AID-mediated mutations outside of immunoglobulin gene locus are involved in the oncogenic transformation of B lymphocytes. Besides its role in B cell lymphomagenesis, AID was recently proposed to play a key role in different human cancers linked to chronic inflammation, or in cancers associated with infectious agents. We first confirmed that both Tax+ and HTLV-1-infected T-cell lines, but not uninfected T cells expressed *aid* mRNA as well as AID protein. We further demonstrated that, primary CD4+ T cells and MOLT-4 T-cell line transduced with lentiviral vector expressing Tax expressed high level of AID. More importantly, we also observed a high level of *aid* in splenic T lymphoma cells obtained from HTLV-1-infected humanized Rag2^-/-^gamma c^-/-^ mice that have developed lymphomas. We demonstrate that AID up-regulation in T cells is associated with DNA damage accumulation. Finally, inhibiting AID expression by small hairpin RNA strategy strongly decreases Tax-induced DNA damages. Altogether our data strongly suggest that AID is involved in DNA damages and genomic instability of HTLV-1-infected T-cells.

